# Time trends in socioeconomic inequalities in cancer mortality: results from a 35 year prospective study in British men

**DOI:** 10.1186/1471-2407-14-474

**Published:** 2014-06-30

**Authors:** Sheena E Ramsay, Richard W Morris, Peter H Whincup, Anna Olia Papacosta, Lucy T Lennon, Sasiwarang Goya Wannamethee

**Affiliations:** 1 Department of Primary Care & Population Health, UCL, London, UK; 2 Division of Population Health Sciences and Education, St George’s University of London, London, UK; 3 Department of Primary Care & Population Health, UCL Medical School, Royal Free Campus, Rowland Hill Street, London NW3 2PF, UK

**Keywords:** Socioeconomic inequalities, Cancer mortality, Trends

## Abstract

**Background:**

Socioeconomic inequalities in cancer mortality in Britain have been shown to be present in the 1990s and early 2000s. Little is known about on-going patterns in such inequalities in cancer mortality. We examined time trends in socioeconomic inequalities in cancer mortality in Britain between 1978 and 2013.

**Methods:**

A socially representative cohort of 7489 British men with data on longest-held occupational social class, followed up for 35 years, in whom 1484 cancer deaths occurred.

**Results:**

The hazard ratio for cancer mortality for manual vs. non-manual social classes remained unchanged; among men aged 50–59 years it was 1.62 (95%CI 1.17–2.24) between 1980–1990 and 1.65 (95%CI 1.14–2.40) between 1990–2000. The absolute difference (non-manual minus manual) in probability of surviving death from cancer to 70 years remained at 3% over the follow-up. The consistency of risks over time was similar for both smoking-related and non-smoking related cancer mortality.

**Conclusion:**

Socioeconomic inequalities in cancer mortality in Britain remain unchanged over the last 35 years and need to be urgently addressed.

## Background

Cancer remains a leading cause of mortality in the UK, accounting for 157, 000 cancer deaths (28% of all deaths) in 2010 [1]. Socioeconomic inequalities in cancer mortality and survival in the UK are well documented [2-8], with higher cancer mortality rates in lower socioeconomic groups than in higher ones. Although survival rates for most cancers have improved in the UK, socioeconomic inequalities in survival are known to persist in England, such that those from lower compared to higher socioeconomic groups have an increased risk of cancer mortality [9]. Different factors contributing to these inequalities are socioeconomic differences in risk factors (such as tobacco), stage of diagnosis and access to treatment [10-13]. Previous findings have shown little reduction in inequalities in cancer survival England between 1996 and 2006 [7].

Recent public health policies in the UK have aimed at reducing socioeconomic inequalities in cancer mortality. The NHS Cancer Plan (2000) for England outlined a strategy to improve cancer survival, with reducing socioeconomic inequalities in cancer mortality as one of its main aims [14]. More recently through the launch of the National Cancer Equality Initiative (NCEI), there has been a greater focus on specifically addressing the issue of health inequalities in cancers [9]. In order to inform these on-going policy efforts, a better understanding and monitoring patterns of changes or trends in inequalities in cancers over time is important to achieve a reduction in these inequalities. Therefore, in this paper, we aim to investigate whether socioeconomic differences in cancer mortality have persisted between 1978–80 and 2013 in a representative cohort of British men. Given the recent declines in the UK in smoking [15], a leading risk factor for cancer [16], which is also strongly socioeconomically patterned [17], we were also interested in assessing changes in socioeconomic inequalities separately for smoking and non-smoking related cancer mortality. We have previously demonstrated that inequalities in all-cause and coronary disease mortality (another leading cause of death) have not narrowed in Britain [18], – in this paper we investigate the same issue for cancer mortality.

## Methods

The British Regional Heart Study is a prospective study comprising a socially and geographically representative sample of 7735 men initially examined in 1978–80 when aged 40–59 years, drawn from one general practice in each of 24 towns representing all major British regions [19]. All men provided written informed consent to the investigations, carried out in accordance with the Declaration of Helsinki. This study was approved by the National Research Ethics Service (NRES) Committee, London Central region. The study was initiated originally to understand geographical variations in cardiovascular disease mortality in Britain [20]. Cohort participants have been followed-up since for morbidity through two-yearly reviews of general practitioner (primary care physician) records. Data on mortality have been obtained through the established procedure of ‘flagging’ participants with the National Health Service Central Register; contact was successfully maintained with >98% of study participants. Follow-up of the participants has also been through postal questionnaires to collect information on general health outcomes. Cancer mortality was ascertained from death certificates with malignant neoplasms identified as the underlying cause of death (*International Classification of Diseases*, 9th revision (ICD-9) ICD 140–208); smoking-related cancer deaths included cancers of the lip, tongue, oral cavity and larynx (ICD codes 140, 141, 143–149), oesophagus (ICD 150), pancreas (ICD 157), respiratory tract (ICD 160–163), bladder (ICD 188), kidney (ICD 189), acute myeloid leukemia (ICD 208.0) and stomach (ICD 151.0; excluding noncardia); [16] all other malignant neoplasms were classified as non-smoking related cancers for the analysis.

Socioeconomic position was based on occupational social class. The longest-held occupation of subjects at study entry (aged 40–59 years) was used to define social class using the Registrar Generals’ Social Class Classification – I (professionals, e.g. physicians, engineers), II (managerial, e.g. teachers, sales managers), III non-manual (semi-skilled non-manual, e.g. clerks, shop assistants), III manual (semi-skilled manual, e.g. bricklayers), IV (partly skilled, e.g. postmen) and V (unskilled, e.g. porters, general labourers) [21]. Information on social class was not available for 15 subjects. Men with the longest-held occupation in the armed forces were excluded from the analyses [231 at baseline (3%)]. Social classes I, II and IIInon-manual were grouped as ‘non-manual’ while social classes IIImanual, IV and V were grouped as ‘manual’ to provide a single overall summary of social inequalities and their trends [18]. This occupational social class measure has been previously used to investigate socioeconomic inequalities in mortality, cardiovascular disease, disability, and trends in inequalities in all-cause and coronary disease in this cohort [18,22,23], and other studies [24-28].

Statistical analysis: Social classes were combined into two groups of non-manual (social class I, II, III non-manual) and manual (III manual, IV and V). Cox’s proportional hazard model was used to assess the relation of social class with all-cancer and smoking-related cancer mortality. Age-adjusted hazard ratios (HRs) with 95% confidence intervals (CI) were calculated for the social class groups using non-manual social classes as the reference category. We examined trends in inequalities in all-cancer and smoking-related cancer mortality over the follow-up time of 35 years from baseline until July 2013. The follow-up time was divided into three calendar periods starting from baseline in 1978–80: 0–10 years (1978–80 to 1988–90), 10–20 years (1988–90 to 1998–2000), and 20–35 years (1998–2000 to 2008–2013). Baseline age was divided into two groups of 40–49 and 50–59. HRs with 95% CI (relative risks) comparing manual with non-manual groups for all-cancer and smoking-related cancer mortality were calculated overall for the two age groups and three calendar periods, and for each age group within each time period. Cox models included effects of age, period, social class, and social class*period interaction (to ascertain whether the social class effect changed over calendar time). To estimate the overall change in relative difference in non-manual vs. manual groups, we calculated the ratio of HRs (or the change in hazard ratio) per year over the entire 35-year calendar period from the Cox regression model estimates – this provided an estimate of the change in hazard ratio (or relative risk) over the entire follow-up period. Similar analyses have been used in the cohort to investigate trends over time in inequalities in all-cause and coronary disease mortality [18].

Rates of death from all-cancer and smoking-related cancer mortality were estimated to ascertain the absolute difference in cancer-free survival between manual and non-manual groups according to the age groups and calendar periods described above. Survival probability to age 70 years was calculated using Cox models for the non-manual and manual groups to estimate the absolute social class difference in survival. Most analyses were carried out using SAS version 9.3; analyses examining social class*age and social class*period interactions were carried out with STATA version 12.

## Results

Results are based on 7489 men aged 40–59 years at baseline followed up for 35 years (193, 155 person years). During this period, 4627 deaths occurred; 1484 deaths were from cancers, of which 734 were smoking-related cancer deaths. Overall HRs comparing manual with non-manual social classes were 1.35 (95% CI 1.21, 1.50) for mortality from all cancers, 1.53 (95% CI 1.32, 1.79) for smoking-related cancers, and 1.20 (95% CI 1.03, 1.38) for non-smoking related cancers. Table 1 presents descriptive characteristics of the cohort.

**Table 1:** Descriptive characteristics of a cohort of British men aged 40–59 years in 1978-80

	**Overall**	**Non-manual group**	**Manual group**
Age (years) – mean (SD)	50 (5.8)	50 (5.8)	50.5 (5.8)
Body mass index (kg/m^2^) – mean (SD)	25.5 (3.2)	25.3 (2.9)	25.6 (3.4)
Systolic blood pressure (mmHg) – mean (SD)	145 (20.9)	143 (20.6)	147 (21)
Current smoking n (%)	3046 (41%)	904 (30%)	2142 (48%)
Moderate/ heavy drinking – n (%)	2787 (37%)	895 (29%)	1892 (43%)
Physical inactivity – n (%)	2928 (40%)	1009 (33%)	1919 (44%)

Table 2 presents age-adjusted HRs for all-cancer, smoking-related and non-smoking related cancer mortality comparing manual with non-manual groups, according to different calendar periods from baseline and two age groups. Changes in relative hazard over calendar time, independent of age, can be observed in Table 2 by following the HRs horizontally across the rows. For example, among men aged 50–59 years the HR for all-cancer mortality was 1.62 (95% CI 1.17, 2.24) in the first 10-year period, and 1.65 (95% CI 1.14, 2.40) for men aged 50–59 years in the next 10-year period; the corresponding HR for smoking-related cancer mortality declined slightly from 1.83 (95% CI 1.18, 2.83) to 1.58 (95% CI 0.96, 2.61), and remained non-significant for non-smoking related cancer mortality - 1.40 (95% CI 0.87, 2.25) and 1.74 (95% CI 0.99, 3.04) respectively. A formal analysis of the social class*period interaction extending over the follow-up period and for all age groups, showed that over a 35-year calendar period, the change in hazard ratio per year comparing manual vs. non-manual groups was 0.99 (95% CI 0.98-1.00, p = 0.09), for all-cancer mortality, 0.99 (95%CI 0.97-1.01, p = 0.17) for smoking-related cancer mortality, and 0.99 (95% CI 0.98, 1.01, p = 0.50) for non-smoking related cancer mortality. These estimates compare the change in hazard ratio (or relative risk) for non-manual vs. manual groups over time. Thus, these estimates indicate that there was no evidence for change in the relative social class differences over the follow-up period.

**Table 2:** **Age-adjusted hazard ratios (95**% **confidence interval) for cancer mortality comparing manual versus non-manual social classes in a cohort of British men followed-up for 35 years**

**Age (years)**	**0-10 years 1978-80 to 1988-90**	**10-20 years 1988-90 to 1998-2000**	**20-35 years 1998-2000 to 2008-2013**
40-49			
N	3515		
All-cancer mortality	1.11 (0.64, 1.93)		
Smoking-related cancer mortality	1.32 (0.55, 3.14)		
Non-smoking-related cancer mortality	0.98 (0.48, 2.02)		
50-59			
N	3764	3364	
All-cancer mortality	1.62 (1.17, 2.24)	1.65 (1.14, 2.40)	
Smoking-related cancer mortality	1.83 (1.18, 2.83)	1.58 (0.96, 2.61)	
Non-smoking-related cancer mortality	1.40 (0.87, 2.25)	1.74 (0.99, 3.04)	
60-69			
N		3227	3027
All-cancer mortality		1.39 (1.10, 1.75)	1.27 (1.04, 1.55)
Smoking-related cancer mortality		1.61 (1.17, 2.21)	1.21 (0.89, 1.63)
Non-smoking-related cancer mortality		1.16 (0.83, 1.63)	1.32 (1.01, 1.73)
70-79			
N			2315
All-cancer mortality			1.23 (1.01, 1.51)
Smoking-related cancer mortality			1.73 (1.25, 2.38)
Non-smoking-related cancer mortality			0.98 (0.75, 1.29)
Overall (all ages)			
All-cancer mortality	1.48 (1.12, 1.95)	1.44 (1.19, 1.75)	1.26 (1.10, 1.46)
Smoking-related cancer mortality	1.72 (1.16, 2.54)	1.56 (1.20, 2.03)	1.44 (1.16, 1.78)
Non-smoking-related cancer mortality	1.26 (0.85, 1.86)	1.30 (0.98, 1.74)	1.14 (0.95, 1.38)

The corresponding absolute differences between non-manual and manual groups in rates of all-cancer, smoking-related and non-smoking related cancer mortality are presented in Table 3. Absolute differences (non-manual–manual) at different calendar periods can be observed by following comparable age groups horizontally across the rows. For example, the absolute social class difference (per 1000 person years) in all-cancer mortality for 50–59 year old men was 2.29 in the first 10-year period and 1.88 in the next 10-year period. Similarly, the absolute difference (per 1000 person years) in smoking-related cancer mortality for 50–59 year old men was 1.61 in the first 10-year period and 1.00 in the next 10-year period; and 0.70 and 0.95 respectively for non-smoking related cancer mortality. An analysis estimating probabilities over a 30-year period from a Cox model showed that the absolute social class difference in the probability of survival to 70 years from all-cancer mortality was 3% in the first 10-year period as well as the last 10-year period. Similarly, the absolute social class difference in probability of survival to 70 years was 2% for smoking-related cancers, and 1% for non-smoking related cancers both in the first 10-year and last 10-year period.

**Table 3:** Rate per 1000 person years and absolute risk of cancer mortality comparing manual versus non-manual social classes in a cohort of British men followed-up for 35 years

**Age (years)**	**0-10 years**			**10-20 years**			**20-35 years**		
	**1978-80 to 1988-90**			**1988-90 to 1998-2000**			**1998-2000 to 2008-2013**		
	**Non-manual**	**Manual**	**Difference**	**Non-manual**	**Manual**	**Difference**	**Non-manual**	**Manual**	**Difference**
			**Manual – non-manual**			**Manual – non-manual**			**Manual – non-manual**
40-49									
All-cancer mortality	1.42	1.57	0.16						
Smoking-related cancer mortality	0.54	0.71	0.17						
Non-smoking-related cancer mortality	0.88	0.86	−0.01						
50-59									
All-cancer mortality	3.70	5.99	2.29	2.90	4.78	1.88			
Smoking-related cancer mortality	1.85	3.46	1.61	1.56	2.56	1.00			
Non-smoking-related cancer mortality	1.78	2.48	0.70	1.27	2.22	0.95			
60-69									
All-cancer mortality				9.30	12.77	3.47	9.55	11.96	2.42
Smoking-related cancer mortality				4.65	7.49	2.84	4.33	5.22	0.89
Non-smoking-related cancer mortality				4.57	5.22	0.65	5.22	6.75	1.53
70-79									
All-cancer mortality							15.92	19.29	3.37
Smoking-related cancer mortality							5.68	9.69	4.01
Non-smoking-related cancer mortality							10.14	9.60	−0.54
Overall (all ages)									
All-cancer mortality	2.53	3.87	1.34	5.85	8.55	2.70	11.74	14.61	2.87
Smoking-related cancer mortality	1.18	2.14	0.96	3.04	4.91	1.87	4.76	6.83	2.07
Non-smoking-related cancer mortality	1.32	1.70	0.39	2.74	3.61	0.87	6.95	7.78	0.83

## Discussion

This study in a British cohort of men followed-up from middle-age shows that socioeconomic inequalities in cancer mortality have persisted from 1978–80 until the present. There was no evidence that the increased risk of all-cancer, smoking-related and non-smoking related cancer mortality in lower socioeconomic groups had changed over the three decades of follow-up. The absolute socioeconomic difference in cancer mortality rates between social classes also remained unchanged.

Our study comprises a socially and geographically representative cohort of men from across Britain. The high follow-up rate (>98%) has enabled us to investigate inequalities in cancer mortality over a 35-year period. Our measure of socioeconomic position (non-manual/ manual social class), based on the longest held occupation recorded at baseline could be defined for nearly all study participants. This social class measure remained consistent for almost all subjects throughout the study; over a 20 year period only 8% of participants changes their social class status under this definition [23]. Therefore, this measure provides a stable and well-established marker of social class. However, the cohort comprised men, mostly Caucasian. Therefore, generalizability of findings to women and other ethnic groups may be limited. However, our results are broadly similar to previous reports on trends in inequalities in cancer [5,7]. Our overall results were also similar (no change in inequalities in cancer mortality) even when using a more detailed social stratification comparing social classes I and II with social classes IV and IV. A limitation of our study is that precise dates of cancer diagnosis were not available for some cases, particularly during the initial follow-up of the cohort; this implied that we were unable to explore inequalities in survival since diagnosis of cancer due to imprecise data on exact date of diagnosis of cancers for some cases. Nevertheless, the inequalities in cancer mortality observed in our results are likely to be due to differences in cancer survival since we observed no evidence of social class differences in non-fatal cancers (results not presented). Another issue with longitudinal analyses as in this study is that of competing risk of mortality from other major causes of death (for example coronary heart disease) during follow-up. We have observed similar persisting inequalities for coronary disease and all-cause mortality in our cohort [18]. Moreover, competing risk, if anything, is likely to underestimate the social class difference observed in cancer mortality, since manual (or lower) social class groups are more likely to die of other causes such as coronary disease. However, this impact of competing risk is likely to be present throughout the follow-up period and therefore, unlikely to affect the overall pattern in inequalities observed in the study, which is the focus of this paper.

Previous studies using data from Cancer Registries in England and Wales have reported that inequalities in cancer survival remained unchanged between 1986 and 2006 [5,7]. Our results on trends in inequalities extend these findings to 2013, and also show that both the relative and absolute socioeconomic differences in cancer mortality have remained substantially unchanged. This pattern observed also for smoking-related cancers could reflect the evidence that although smoking rates have declined overall, socioeconomic differences in smoking continue to persist with higher smoking rates in lower socioeconomic groups [17,29]. Although, the purpose of the present study was not to investigate possible factors underlying inequalities in cancer mortality, previous studies show that other possible factors contributing to these persisting inequalities are socioeconomic differences in stage of diagnosis, access to treatment, or patient-level factors including nutrition and health-seeking behaviours [2]. Policy efforts through programmes such as the National Cancer Equality Initiative are needed to address this trend of persisting socioeconomic inequalities in cancer in the UK. These programmes aimed at reducing inequalities in cancer mortality will need continuous evaluation to reduce cancer mortality in lower socioeconomic groups. Further research is also needed to provide evidence for effective public health interventions to reduce socioeconomic inequalities in cancer mortality.

## Conclusions

In summary, this paper shows that socioeconomic inequalities in cancer mortality have persisted over the last 35 years in Britain. Concerted efforts to address the continuing increased risk of cancer mortality in lower socioeconomic groups needs to be addressed.

## Abbreviations

CI: Confidence interval; ICD: International Classification of Diseases.

## Competing interests

The authors declare that they have no competing interest.

## Authors’ contribution

SER, PHW, SGW and RWM developed the original idea for the paper, and SER wrote the first draft and performed the analyses. SER, PHW, SGW, RWM, LTL and AOP contributed to the study design and collation of data. All authors contributed to interpretation of data and the final version of the manuscript, and all are guarantors.

## Pre-publication history

The pre-publication history for this paper can be accessed here:

http://www.biomedcentral.com/1471-2407/14/474/prepub

## References

[B1] Cancer Research UK Cancer Incidence and Mortality in the UK for the 20 Most Common Cancers 2012 UK, London: Cancer Research

[B2] Woods LM Rachet B Coleman MP Origins of socio-economic inequalities in cancer survival: a review Ann Oncol 2006 17 5 19 16143594 10.1093/annonc/mdj007

[B3] Davey Smith G Hart C Blane D Hole D Adverse socioeconomic conditions in childhood and cause specific adult mortality: prospective observational study BMJ 1998 316 1631 1635 9603744 10.1136/bmj.316.7145.1631 PMC28561

[B4] Berglund A Lambe M Lüchtenborg M Linklater K Peake MD Holmberg L Møller L Social differences in lung cancer management and survival in South East England: a cohort study BMJ Open 2012 2 e001048 10.1136/bmjopen-2012-001048 PMC3367157 22637374

[B5] Coleman MP Rachet B Woods LM Mitry E Riga M Cooper N Quinn MJ Brenner H Esteve J Trends and socioeconomic inequalities in cancer survival in England and Wales up to 2001 Br J Cancer 2004 90 1367 1373 15054456 10.1038/sj.bjc.6601696 PMC2409687

[B6] Ellis L Coleman MP Rachet B How many deaths would be avoidable if socioeconomic inequalities in cancer survival in England were eliminated? A national population-based study, 1996–2006 Eur J Cancer 2012 48 270 278 22093945 10.1016/j.ejca.2011.10.008

[B7] Rachet B Ellis L Maringe C Chu T Nur U Quaresma M Shah A Walters S Woods L Forman D Coleman MP Socioeconomic inequalities in cancer survival in England after the NHS cancer plan Br J Cancer 2010 103 446 453 20588275 10.1038/sj.bjc.6605752 PMC2939774

[B8] Sharpe KH McMahon AD Raab GM Brewster DH Conway DI Association between socioeconomic factors and cancer risk: a population cohort study in Scotland (1991–2006) PLoS One 2014 9 e89513 24586838 10.1371/journal.pone.0089513 PMC3937337

[B9] National Cancer Equality Initiative Reducing Cancer Inequality: Evidence, Progress and Making it Happen 2010 London: Department of Health

[B10] Braaten T Weiderpass E Kumle M Lund E Explaining the socioeconomic variation in cancer risk in the Norwegian women and cancer study Cancer Epidemiol Biomarkers Prev 2005 14 2591 2597 16284383 10.1158/1055-9965.EPI-05-0345

[B11] Schrijvers CTM Mackenbach JP Lutz J Quinn MJ Coleman MP Deprivation, stage at diagnosis and cancer survival Int J Cancer 1995 63 324 329 7591225 10.1002/ijc.2910630303

[B12] Schwartz K Crossley-May H Vigneau F Brown K Banerjee M Race, socioeconomic status and stage at diagnosis for five common malignancies Cancer Causes Control 2003 14 761 766 14674740 10.1023/a:1026321923883

[B13] Quaglia A Lillini R Mamo C Ivaldi E Vercelli M Socio-economic inequalities: a review of methodological issues and the relationships with cancer survival Crit Rev Oncol Hematol 2013 85 266 277 22999326 10.1016/j.critrevonc.2012.08.007

[B14] Department of Health The NHS Cancer Plan: a Plan for Investment, a Plan for Reform 2000 London: Department of Health

[B15] Office for National Statistics Smoking (General Lifestyle Survey Overview - a Report on the 2011 General Lifestyle Survey) General Lifestyle Survey, 2011 Release 2013 London: Office for National Statistics

[B16] U.S. Department of Health and Human Services The Health Consequences of Smoking: a Report of the Surgeon General 2004 Atlanta, Georgia: U.S. Department of Health and Human Services, Centers for Disease Control and Prevention, National Center for Chronic Disease Prevention and Health Promotion, Office on Smoking and Health

[B17] Ramsay SE Whincup PH Hardoon SL Lennon LT Morris RW Wannamethee SG Social class differences in secular trends in established coronary risk factors over 20 years: a cohort study of British men from 1978–80 to 1998–2000 PLoS One 2011 6 e19742 21603647 10.1371/journal.pone.0019742 PMC3094451

[B18] Ramsay SE Morris RW Whincup PH Lennon LT Wannamethee SG Are social inequalities in mortality in Britain narrowing? Time trends from 1978 to 2005 in a population-based study of older men J Epidemiol Community Health 2008 62 75 80 18079337 10.1136/jech.2006.053207 PMC2709224

[B19] Walker M Whincup PH Shaper AG The British Regional Heart Study 1975–2004 Int J Epidemiol 2004 33 1185 1192 15319395 10.1093/ije/dyh295

[B20] Shaper AG Pocock SJ Walker M Cohen NM Wale CJ Thomson AG British Regional Heart Study: cardiovascular risk factors in middle-aged men in 24 towns BMJ 1981 283 179 186 6789956 10.1136/bmj.283.6285.179 PMC1506709

[B21] Classification of Occupations 1970 1970 London: HM Stationary Office

[B22] Ramsay SE Whincup PH Morris RW Lennon LT Wannamethee SG Extent of social inequalities in disability in the elderly: results from a population-based study of British Men Ann Epidemiol 2008 18 896 903 19041588 10.1016/j.annepidem.2008.09.006 PMC2728204

[B23] Emberson JR Whincup PH Morris RW Walker M Social class differences in coronary heart disease in middle-aged British men: implications for prevention Int J Epidemiol 2004 33 289 296 15082628 10.1093/ije/dyh006

[B24] Smith GD Hart C Watt G Hole D Hawthorne V Individual social class, area-based deprivation, cardiovascular disease risk factors, and mortality: the Renfrew and Paisley Study J Epidemiol Community Health 1998 52 399 405 9764262 10.1136/jech.52.6.399 PMC1756721

[B25] Brown J Harding S Bethune A Rosato M Incidence of Health of the Nation Cancers by Social Class Population Trends 1997 90 40 47 9449159

[B26] Hart CL Hole DJ Gillis CR Smith GD Watt GC Hawthorne VM Social class differences in lung cancer mortality: risk factor explanations using two Scottish cohort studies Int J Epidemiol 2001 30 268 274 11369726 10.1093/ije/30.2.268

[B27] Shohaimi S Welch A Bingham S Luben R Day N Wareham N Khaw K-T Area deprivation predicts lung function independently of education and social class Eur Respir J 2004 24 157 161 15293619 10.1183/09031936.04.00088303

[B28] Galobardes B Lynch J Davey Smith G Measuring socioeconomic position in health research Br Med Bull 2007 81–82 21 37 10.1093/bmb/ldm001 17284541

[B29] Ferrie JE Shipley MJ Davey Smith G Stansfeld SA Marmot MG Change in health inequalities among British civil servants: the Whitehall II study J Epidemiol Community Health 2002 56 922 926 12461113 10.1136/jech.56.12.922 PMC1757010

